# First person – Mary Salcedo

**DOI:** 10.1242/bio.048199

**Published:** 2019-10-15

## Abstract

First Person is a series of interviews with the first authors of a selection of papers published in Biology Opens, helping early-career researchers promote themselves alongside their papers. Mary Salcedo is first author on ‘Computational analysis of size, shape and structure of insect wings’, published in BiO. Mary conducted the research described in this article while a Graduate Student in L. Mahadevan's lab at Harvard University, Cambridge, USA. She is now a NSF Postdoctoral Researcher in Biology in the lab of Jake Socha at Virginia Tech, USA, investigating insect wing shapes, venation patterns and circulation within the wings.


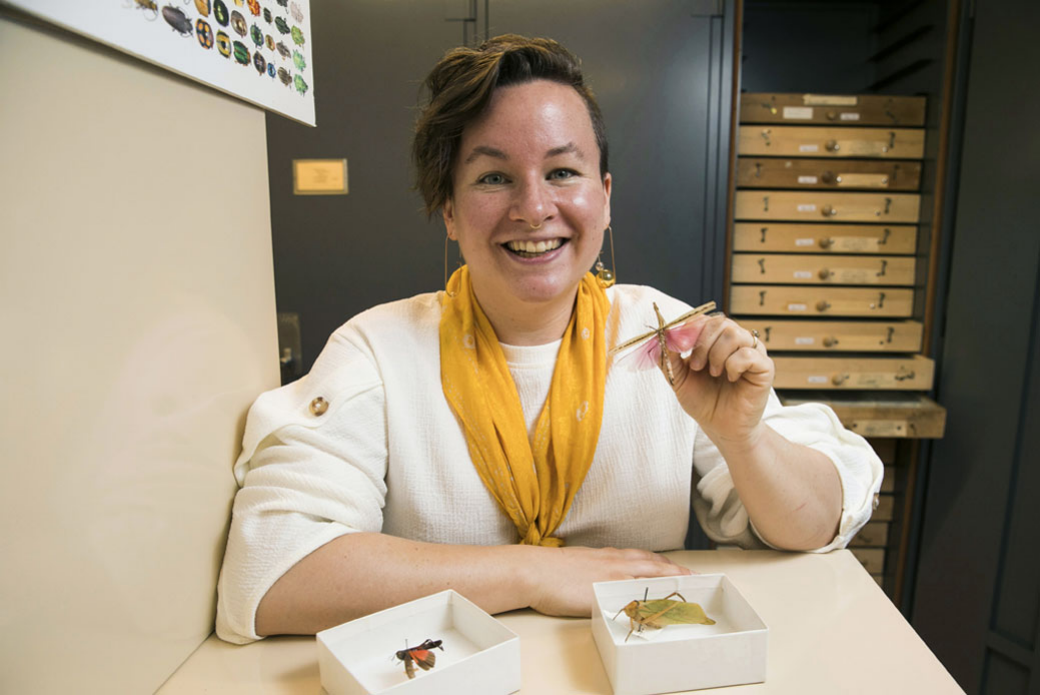


**Mary Salcedo**

**What is your scientific background and the general focus of your lab?**

By training, I'm an insect biomechanist, but perhaps functional morphologist is more accurate. I've studied how insects fly, how their muscles move, how they breathe and circulate hemolymph. My PhD looked into their wing structure at several levels: external, internal and global. Externally, I looked at how wing shapes differ between species and how we might compare them. Within the insect wing vein, I studied how hemolymph (insect blood) is transported across the wing. Overall, I looked at how an insect's multiple hearts contribute to internal circulation.

“We are one of the first to put all of these wings from many different species on the same geometric space.”

**How would you explain the main findings of your paper to non-scientific family and friends?**

Insect wing diversity is so vast, but there are common shapes between wings that can tell us about how they evolved such complex structures. Identifying these common geometries is difficult! So, before any analysis was done, we created a method to process our collected 789 insect wings. First, we collected real insect wings and detailed illustrations from entomology texts and scanned them, resulting in high-resolution images. Secondly, we ‘segmented the image’, meaning we found the digital skeleton of the wing (its venation pattern). Once we had that, we were able to measure simple geometries of each insect wing and compare wildly different wings. I tell folks to imagine a dragonfly wing and a fruit fly wing. The dragonfly wing is large, and full of veins, and hundreds of small ‘domains’, which are just the various shapes within a wing. The fruit fly wing is tiny and has a sparse venation, where its wing shapes within the wing are relatively large. We measured wing perimeter and internal wing structure (we added up all the lengths of vein inside a wing), and normalized them, meaning we can compare wings no matter their size. We also measured all of the shapes within a wing (domains) and asked (1) how circular are these shapes and (2) how much space do they take up in a wing? So, with all these measurements, we made simple spaces of shape relationships (we called them morphospaces), which are just representations of all our wing measurements. Some of our findings may seem obvious, for example, we found that large shapes take up more wing space, and that wings with lots of venation will have many small, circular shapes. However, this method and the measurements we made are from a broad sweep of hundreds of insect wings, and we are one of the first to put all of these wings from many different species on the same geometric space.

**What are the potential implications of these results for your field of research?**

We have barely scratched the surface of insect wing diversity. Here, we've provided a comprehensive dataset of wings across the insect phylogeny, their data (segmented image), and tools to add to the analysis of these wings and to view them in simple morphospaces. This is an exciting swath of data, and I'm looking forward to finding out how a phylogeneticist might use these relationships. Not only that, but I feel this area is ripe for questions – thinking about how wing flexibility and damage affect geometries, how environment and specific ecologies may influence overall wing shape, or even how behaviors drive wing shape over time. We hope that scientists will use our method to not only add to insect wing data, but to ask even more questions.

**What has surprised you the most while conducting your research?**

Diving into resources, I was generally surprised by the richness of older entomology texts. Wandering between the stacks of the Ernst Mayr Library, I would pull down book after book, finding detailed and accurate drawings of insect wings. While scanning of scientific texts has made an incredible amount of literature available online (we frequently searched the Biodiversity Heritage Library) many texts remain untouched. This project highlighted the importance of that movement to put literature online. There are endless rich data in those texts!

“Wandering between the stacks of the Ernst Mayr Library, I would pull down book after book, finding detailed and accurate drawings of insect wings.”

**What, in your opinion, are some of the greatest achievements in your field and how has this influenced your research?**

This is difficult! I would say though, in the field of insect biomechanics, my most referenced texts were from a co-advisor and mentor, Dr Stacey Combes. Her work inspired an interest in wing venation and the myriad of patterns found across insects. Her thesis sampled wing flexibility across the phylogeny, and was one of the first studies to measure stiffness and flexibility of insect wings. I would say it has influenced most projects involving flying micro-robotics or insect flight.

**Figure:**
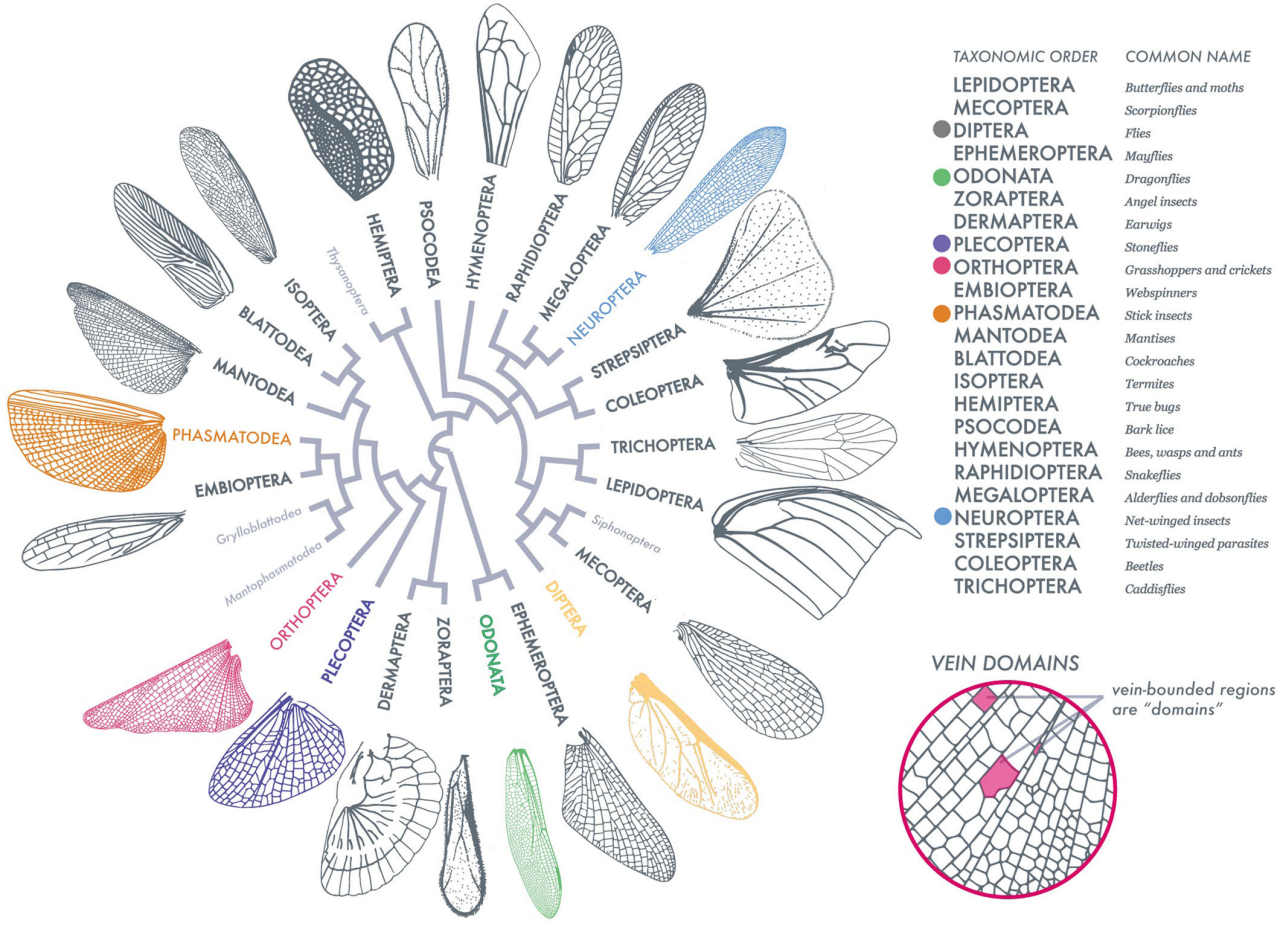
**Insect wing venation patterns, wing contour and shapes of vein-bounded domains.** Adapted from Misof et al. (2014).

**What changes do you think could improve the professional lives of early-career scientists?**

Early-career scientists need much more support than they are currently receiving. This support needs to be financial, through mentorship, and through resources at the university. Most universities do not provide enough career (finding jobs), financial or camaraderie (unity among post docs) support to these scientists. I hear countless stories of how post-docs feel lost or unimportant at their university. Being highly trained scientists, we need more honest and open access to these things. In my current position, I am grateful to have many of these things, but I find at the university level, no one knows what I am, what I do, and most offices have a difficult time giving me access to buildings/resources.

**What's next for you?**

I recently moved to Virginia Tech to start an NSF-funded post-doctoral research fellowship in the Socha Lab. This lab works on a variety of biomechanics projects, such as how snakes glide, how insects breathe and move hemolymph, how frogs can run on water, and so much more. I'll be looking in depth at how pumping organs, specifically the wing hearts, contribute to circulation in the wings and overall flow within an insect. I'm looking forward to an upcoming trip at the Advanced Photon Source to image insects.
